# Invasive Fungal Infections in Persons Living with HIV in an Amazonian Context: French Guiana, 2009–2019

**DOI:** 10.3390/jof7060421

**Published:** 2021-05-27

**Authors:** Laurène Cachera, Antoine Adenis, Basma Guarmit, Sébastien Rabier, Pierre Couppié, Felix Djossou, Loïc Epelboin, Alessia Melzani, Philippe Abboud, Denis Blanchet, Magalie Demar, Kinan Drak Alsibai, Mathieu Nacher

**Affiliations:** 1 Université Paris Descartes, Sorbonne Paris Cité, Paris 75005, France; laurene.cachera@gmail.com; 2 Centre d’Investigation Clinique, Inserm CIC 1424, Centre Hospitalier de Cayenne, 97300 Cayenne, French Guiana; antoine.adenis@ch-cayenne.fr (A.A.); loic.epelboin@ch-cayenne.fr (L.E.); 3 Département Formation Recherche, Université de Guyane, 97300 Cayenne, French Guiana; pierre.couppie@ch-cayenne.fr (P.C.); felix.djossou@ch-cayenne.fr (F.D.); magalie.demar@ch-cayenne.fr (M.D.); 4 Corevih Guyane, Centre Hospitalier de Cayenne, 97300 Cayenne, French Guiana; basma.guarmit@gmail.com (B.G.); sebastien.rabier@ch-cayenne.fr (S.R.); 5 Service de Dermatologie, Centre Hospitalier de Cayenne, 97300 Cayenne, French Guiana; 6 Service des Maladies Infectieuses et Tropicales, Centre Hospitalier de Cayenne, 97300 Cayenne, French Guiana; allessia.melzani@ch-cayenne.fr (A.M.); philippe.abboud@ch-cayenne.fr (P.A.); 7 Laboratoire de Parasitologie et Mycologie, Centre Hospitalier de Cayenne, 97300 Cayenne, French Guiana; denis.blanchet@ch-cayenne.fr; 8 Unité Mixte de Recherche Tropical Biome and Immuno Pathology, Université de Guyane, 97300 Cayenne, French Guiana; 9 Service d’Anatomopathologie, Centre Hospitalier de Cayenne, 97300 Cayenne, French Guiana; kdrak.alsibai@doctor.com

**Keywords:** invasive fungal infections, HIV, histoplasmosis, cryptococcosis, pneumocystosis, Amazon

## Abstract

Although the burden of histoplasmosis in patients with advanced HIV has been the focus of detailed estimations, knowledge about invasive fungal infections in patients living with HIV in an Amazonian context is somewhat scattered. Our goal was thus to adopt a broader view integrating all invasive fungal infections diagnosed over a decade in French Guiana. All patients hospitalized at Cayenne hospital from 1 January 2009 to 31 December 2018 with a proven diagnosis of invasive fungal infection were included (n = 227). *Histoplasmosis* was the most common (48.2%), followed by Cryptococcus infection (26.3%), and pneumocystosis (12.5%). For cryptococcal infection, there was a discordance between the actual diagnosis of cryptococcal meningitis n = (26) and the isolated presence of antigen in the serum (n = 46). Among the latter when the information was available (n = 34), 21(65.6%) were treated with antifungals but not coded as cryptococcocosis. Most fungal infections were simultaneous to the discovery of HIV (38%) and were the AIDS-defining event (66%). The proportion of major invasive fungal infections appeared to remain stable over the course of the study, with a clear predominance of documented *H. capsulatum* infections. Until now, the focus of attention has been histoplasmosis, but such attention should not overshadow other less-studied invasive fungal infections.

## 1. Introduction

Fungal infections (FIs) represent an emerging health issue worldwide, and their contribution to the morbidity and mortality of persons living with HIV (PLWHIV) is still underestimated [1,2]. While the advent of antiretroviral treatments has revolutionized the management of PLWHIV, invasive fungal infections remain a major threat in countries where access to these treatments is less accessible or among persons unaware of their status. Although a decrease in mortality has been described among PLWHIV for *Pneumocystis jirovecii* and cryptococcosis in developed countries [3], mortality due to FIs is still estimated to be at least one million deaths per year worldwide [1]. Indeed, the HIV epidemic is far from being controlled in many parts of the world, particularly in emerging countries where endemic fungal diseases suffer from a lack of knowledge and availability of reliable diagnostic methods [4]. This is particularly the case for histoplasmosis in South America, recognized as one of the first opportunistic infections at the AIDS stage, even before tuberculosis [5,6,7]. Although less salient than mucous candidiases, invasive candidiasis can also be observed in persons with advanced HIV. Azole resistance is an emerging problem, due to frequent exposure to azole anti-fungals and the emergence of non-*albicans* species with lower sensitivity to fluconazole [8].

French Guiana is the only French territory in South America and is the largest region in France. However, it presents climatic, geographical, socio-economic and cultural particularities relative to mainland France. The population was estimated at 280,000 inhabitants in 2018 [9], and is growing rapidly. It is marked by a significant ethnic diversity, one in three inhabitants being of foreign nationality (mainly Brazilians, Surinamese, Haitians, Dominicans). The epidemiology of infections is also particular [10]: the incidence of HIV is the highest in the country (15.4/1000 patients per year) and histoplasmosis is the most frequent opportunistic infection among people living with HIV at the AIDS stage [6], before candidiasis and pneumocystosis. Cryptococcosis appears to have a higher incidence rate than elsewhere in South America or mainland France [11].

Although the burden of histoplasmosis has been the focus of detailed estimations, knowledge about invasive fungal infections in an Amazonian context is somewhat scattered. Our goal was thus to adopt a broader view integrating all invasive fungal infections diagnosed over a decade in French Guiana, using HIV cohort information systems in order to sharpen clinical practices and thus, ultimately, reduce the morbidity and mortality of invasive fungal infection among PLWHIV coming from the Amazonian region.

## 2. Materials and Methods

The study was observational, retrospective, descriptive, and monocentric covering the period from 1 January 2009 to 31 December 2018.

### 2.1. Study Conduct

The source population was represented by all patients hospitalized at one of the 3 hospitals with no age limit, with a proven diagnosis of invasive fungal infection. An invasive fungal infection was defined as fungal infection with deep tissue or blood involvement. This included any episode of invasive fungal infection, diagnosed between 1 January 2009 and 31 December 2018, proven according to the 2008 modified EORTC/MSG criteria [12,13] or by a positive direct examination for *Pneumocystis jirovecii* in indirect immunofluorescence. HIV patients were subsequently identified through the NADIS database. This database was also used to check for antifungal treatment in cases where the laboratory reported a positive sample, but the diagnosis did not mention the diagnosis. Patients with explicit opposition to research were not included.

Probable or possible invasive fungal infections, relapses and/or recurrences of the same previously proven invasive fungal infection, invasive fungal infections in which several *Candida* species were isolated from the same specimen, invasive fungal infections managed on an outpatient basis, blood cultures of *Aspergillus* spp. (most often reflecting contamination) were excluded [3]. The choice was made not to include probable or possible infections in order to limit bias and facilitate comparisons with other studies.

### 2.2. Diagnostic Methods

Invasive fungal infections were diagnosed using routine methods. HIV-infected patients with suspected opportunistic infections were screened for various pathogens, with a core of blood and urine tests, and eventually extensive medical imagery, endoscopies, spinal fluid examinations according to clinical presentation. Direct examinations of fluids or tissues were performed by staining methods (MGG, Gomori Groccot if MGG was negative, and India ink systematically on the CSF), or by indirect immunofluorescence for the detection of *Pneumocystis* spp. (Biorad commercial kit). Cultures were obtained after inoculation on solid Sabouraud/chloramphenicol/gentamicin medium without actidione and incubation at 30 °C and monitored up to 8 weeks for late growth.

Fungal blood cultures were performed on a two-phase Hemoline (Biomerieux) flask until 2015, with incubation at 30 °C, then after 2015 on centrifuged EDTA tube, with inoculation of the buffy coat on BHI liquid medium without antibiotic, and Sabouraud/chloramphenicol/gentamicin solid medium without actidione at 30 °C. BACT/ALERT blood cultures were used in the mycology laboratory and could contribute to the detection of certain fungi. The mycological identification was based on microscopic aspects and was confirmed by MALDI-ToF for all yeasts and sometimes for unusual filamentous fungi. Some strains needed to be sent to the Centre National de Référence des Mycologies Invasives et Antifongiques (CNRMA) (Institut Pasteur—75724 Paris CEDEX 15—France) for biobanking and/or identification, but rarely since the use of MALDI-ToF. The search for cryptococcal antigen in serum, urine, CSF, and/or BAL was performed by a latex agglutination test (Pasteurex–Biorad), or by a rapid immunochromatography test (Immy) from 2013 onwards, since the use of the CrAg Lateral Flow Assay titrations were no longer performed. *Histoplasma* antigen detection, as well as molecular biology techniques (PCR Pneumocystis, Pan Fungal, *Histoplasma*) were not routinely available.

### 2.3. Ethical and Regulatory Aspects

This study complied with the reference methodology N°4 (MR–004), relating to the processing of personal data implemented within the framework of Research Not Involving the Human Person. A commitment of conformity was carried out with the CNIL. The study has been registered in the local General Data Protection Regulation registry (GDRP) according to the regulation (EU) 2016/679 of the European Parliament and of the Council of 27 April 2016 on the protection of natural persons with regard to the processing of personal data and on the free movement of such data, and repealing Directive 95/46/EC.

### 2.4. Statistical Analyses

The final data analysis was conducted using Statistical Software, version 12.0 (STATA) and Microsoft Excel. Qualitative variables were described by proportions and frequencies. Continuous quantitative variables were described by means and standard deviations for variables with a normal distribution or by medians and interquartile ranges for variables with a non-normal distribution. Unconditional logistic regression was used to compare patients with histoplasmosis and other invasive fungal infections. A Hosmer and Lemeshow goodness of fit test was used.

## 3. Results

### 3.1. Characteristics of the Population

The main population characteristics are shown in Table 1. Over the study period, 227 patients living with HIV had a total of 274 invasive fungal infections, which represented for the study period 2.5 invasive fungal infection per 100 person-years. The majority were men (Sex Ratio = 1.7) from border countries (79%), primarily Haiti. Among all patients, 88 (31.2%) were on cotrimoxazole primary prophylaxis overall and among those with pneumocystis 6 (17.1%) had a prescription for it.

### 3.2. Characteristics of Infections

The etiological spectrum of isolated organisms is shown in Table 2. *Histoplasma capsultatum* var. *capsultatum* infections were the most common (48.2%), followed by cryptococcal disease (26.3%), pneumocystosis (12.5%), and invasive candidiasis. There were 31 positive blood cultures for *Candida* spp. In seven cases, the patients were in ICU; overall 5 patients (12%) died within a year. Although, clinical records mentioned invasive candidiasis in only eight cases, 26/31 patients with positive blood cultures were actually treated with antifungals. Among the five that were not treated despite having had a positive culture, all had more than 300 CD4 lymphocytes, one ran away from the hospital, one had a transient moderate neutropenia, one had digestive surgical complications, one had a femoral catheter for dialysis, another had surgery, and for one the medical records contained very little information.

Overall, 86 fungal infections were contemporary of the discovery of HIV (38%), and 147 infections inaugurated the AIDS stage (66%). For *Cryptococcus,* 26 cases of cryptococcal meningoencephalitis were extracted from the clinical databases. However, the laboratory yielded an additional 44 patients with positive antigen detection that had not been coded in the ICD diagnoses in the medical records. We verified in the medical records whether these cryptococcal antigen-positive patients had actually received antifungal treatment; the data was available for 32 patients of whom 21 (65.6%) had received antifungal treatment. After searching in the medical records, the 11 non-treated patients generally had isolated positive results that were not confirmed in subsequent measurements or the electronic medical records were incomplete.

Overall, eight patients of 70 (11.4%) with Cryptococcus infection (four had a positive diagnosis in the laboratory, but the ICD codes were not in the medical records) died within a year after diagnosis; 14/122 (11.5%) patients with histoplasmosis died within a year after diagnosis; 5/34 (12%) patients with invasive candidiasis died within a year; and 1/30 (3.3%) patients died within a year after pneumocystosis.

Compared to other invasive fungal infections, histoplasmosis infections were observed more frequently in patients from Brazil and Surinam (Table 3). There were no statistically significant differences in age, sex, or CD4 nadir between patients with histoplasmosis compared to those without histoplasmosis.

For age at diagnosis, lowest CD4 count, and the delay between the diagnosis of HIV and the diagnosis of the fungal infection, there was no significant difference between the main infections histoplasmosis, cryptococcosis, and pneumocystosis (Kruskal Wallis test, *p* = 0.25).

### 3.3. Temporal Trends

**Figure 1:**
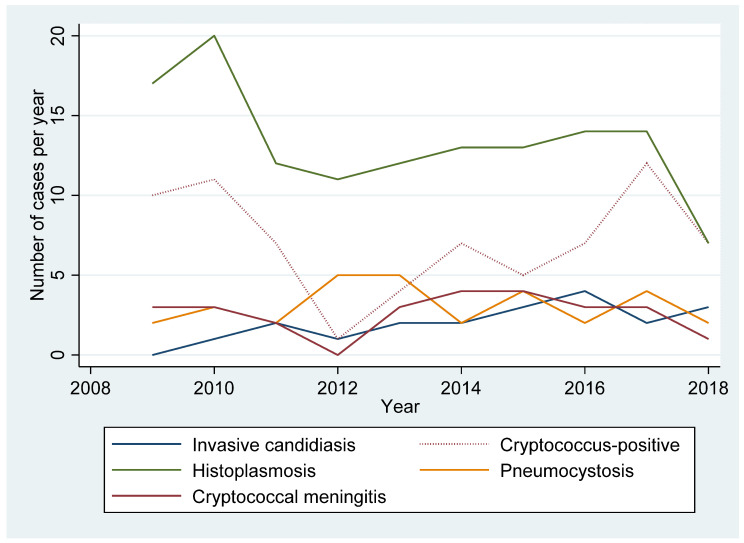
Temporal trends of invasive fungal infections in HIV from 2009 to 2018.

The proportion of major invasive fungal infections appeared to remain stable over the course of the study, with a clear predominance of documented *H. capsulatum* infections (between 10 and 20 cases/year, Figure 1). Figure 1 also distinguishes the clinical diagnoses of cryptococcosis with positive antigenemia only. Overall, estimates of cryptococcosis was far greater that what 10th ICD classification suggested—1.8 times greater (from 26 to 47).

## 4. Discussion

This study is a reminder of the predominant burden of *H. capsulatum* infections among invasive fungal infections of PWHIV. The incidence, in patients with CD4 counts of less than 50/mm^3^, can be as high as 10 per 100 patients-years in French Guiana [6], making it the first opportunistic infection in HIV patients at the AIDS stage [13]. Despite its importance in South America, histoplasmosis remains too often misunderstood and confused with tuberculosis, even though it seems to outnumber tuberculosis in terms of the number of patients affected and mortality [7,14,15]. This can be explained, among other things, by diagnostic difficulties, notably because of the long unavailability of antigenic tests. Although it has been available for more than 30 years in the United States and is a cornerstone in the diagnosis of histoplasmosis [16,17], it remains under-deployed in highly endemic regions, which probably explains the under-estimation of the number of cases diagnosed and the resulting major mortality [18,19]. Thus, in the countries where this technique has been implemented, the number of diagnosed cases has been increased up to 6 times, and the mortality of PLWHA has decreased [20,21]. The relative stability of the annual number of histoplasmosis diagnoses in this study reflects, in any case, the stable and substantial—around 30%—proportion of patients diagnosed with advanced HIV, and therefore, in an endemic context, at risk of developing disseminated histoplasmosis [22], despite a considerable HIV-testing effort in this French region [23]. The greater proportion of Brazilian and Surinamese patients among those with histoplasmosis relative to other invasive fungal infections may have reflected a longer exposure for these nationalities from endemic South America relative to the Caribbean or mainland France or differences in exposure within French Guiana.

Infections related to *Pneumocystis jirovecii* were far behind *H. capsulatum* infections. In mainland France, pneumocystosis remains the main cause of opportunistic infections among PLWHA, on par with tuberculosis [3]. Over two thirds of patients were not receiving cotrimoxazole prophylaxis, so the hypothesis of high primary prophylaxis coverage to explain the difference with mainland France does not seem plausible. This difference in ranking may simply reflect eco epidemiologic differences in an Amazonian context. However, in Cayenne, molecular biology techniques are still not consistently available and physiotherapists are in short supply, hence the quality of expectoration samples may not be as contributive; therefore, it is possible that *Pneumocystis jirovecii* is underestimated in French Guiana.

Cryptococcal infections are a major cause of mortality at the AIDS stage throughout the world. In French Guiana, a retrospective study from 1998 to 2008 revealed a predominance of *C. neoformans var grubii* infections in HIV-infected patients (13/29). All cases of *C. gatii* infection were isolated from non-HIV infected patients. Since 2009, in HIV-infected patients there has been a large majority of *C. neorformans* infections and one *C. gatii* infection. Unfortunately, species diagnosis was almost never available and the proportion of different serotypes is therefore not assessed in this study. Antigen titration was also not available in our records. Here, the laboratory databases yielded far more positive Cryptococcus antigenemia than the number of cryptococcosis reported in the medical records and hospital information system. This discordance suggests that asymptomatic antigenemia was not coded as cryptococcal meningitis, thus suggesting a substantial underestimation of the importance of this fungal infection by information systems based on ICD classifications [14]. Among the eight patients who died within a year of the diagnosis, half had antigen-positive spinal fluid and half had antigen-positive serum. Most of these were laboratory-positive, but uncoded diagnoses were treated with antifungals; however, some were not—usually because they were an isolated finding and other explorations were negative. Hence, for cryptococcal disease, and perhaps for histoplasmosis, the natural disease history of gradual dissemination before patent disease is important to take into account when estimating incidence.

Invasive candidiasis was thought to be a rare complication. However, its total numbers were close to those of pneumocystosis. We could not find evidence of treatment in the electronic records for five non immune-compromised patients with an isolated positive blood culture for *Candida* spp. usually in a context that did not suggest septicemia. The susceptibility profile of isolated *Candida* species was not investigated in this study, which could be of interest, given the emerging problem of antifungal resistance in the world [8]. It is noteworthy that one case of *Candida auris*—an emergent pathogen—was isolated [24].

No proven aspergillosis according to EORTC/MSG criteria was diagnosed in this study. However, this fungus has a ubiquitous distribution that spares neither South America nor PLWHA [1]. Although invasive aspergillosis remains a rare complication in patients with advanced HIV—it usually affects neutropenic patients—its evolution is most often fatal if left untreated. The EORTC criteria may be insufficient to establish a diagnosis in HIV patients, and it has been suggested that invasive aspergillosis remain unrecognized [24,25]. However, in a context where experienced mycologists basically search for any fungal pathogen, we are inclined to conclude that aspergillosis is indeed exceptionally rare among patients with advanced HIV in French Guiana.

This study has limitations. First of all, it was monocentric perhaps not capturing the diversity of clinical presentations in different ecosystems. Clinical data and antifungigrams were not available. Finally, some diagnostic methods were not available during the study period, including molecular biology techniques. However, it was based on accurate microbiological data and therefore provides a reliable idea of the proportion of different invasive fungal infections among PHAs between 2009 and 2019 in French Guiana. It also illustrates that, until now, the focus of attention has been disseminated histoplasmosis, but such polarized attention may have overshadowed other less-studied invasive fungal infections that may require increased vigilance and perhaps an integrated view of invasive fungal infections—their combined and respective importance, their diagnosis in a context of multiple fungal pathogens, and their treatment.

## 5. Conclusions

Invasive fungal infections among PLWHIV in French Guiana remain constantly dominated by *H. capsultatum* infections. In contrast with mainland France, *P. jirovecii* is not the main fungal pathogen. When including results from laboratory databases, cryptococcosis seemed substantially more frequent that what has previously been reported based on clinical reports only, thus representing the main invasive fungal infection after histoplasmosis. Invasive candidiasis was more frequent than expected, but mostly reflected isolated cultures. Overall, in our Amazonian context, invasive fungal infections represent a substantial fraction of opportunistic infections in patients with advanced HIV. Although over half of these are histoplasmosis—a fact that is well known in French Guiana—over 40% are caused by other fungal pathogens, a fact that should kept in mind when exploring patients with advanced HIV in the region.

## Figures and Tables

**Table 1:** Populations characteristics.

	N (%)
Variable	
Male gender, n (%)	160 (62.5%)
Age at diagnostic, median (IQR)	40 (34; 48.8)
Country, n (%) of birth	
France, n (%)	46/227 (20%)
France mainland or Antilles	31
French Guiana	15
Foreign countries, n (%)	171/227 (75.3%)
Latin America	
Brazil	52
Surinam	37
Guyana	13
Venezuela	1
Caribbean	
Haiti	56
Dominican republic	5
St. Lucia	4
Africa	3
NA	38
HIV	
Known HIV status before diagnosis	191/227 (84.1%)
AIDS before diagnostic	62/227 (28%)
Contamination mode	
Heterosexual	206
Homosexual	9
Intraveinous drug use	2
Maternal-fetal infection	7
Transfusion	3
NA	25

The number of patients for whom data were available is indicated, in case of missing data, in the denominator. NA = not available.

**Table 2:** Species distribution.

Fungi	Total
	N = 274
*Histoplasma capsulatum var capsulatum*	133 (48.5%)
*Cryptococcus* spp.	70 (25.5%)
*C. neoformans*	17
*C. neoformans* var *grubii*	1
*C. gattii*	1
NA (positive antigen detection)	54
*Pneumocystis jirovecii*	35 (12.8%)
*Candida* spp.	31 (11.3%)
*Candida albicans*	15
*Candida* non albicans	16
*C. parapsilosis*	9
*C. tropicalis*	2
*C. glabrata*	2
*C. krusei*	1
*C. famata*	1
*C. métapsilosis*	1
*Paracoccidioides brasiliensis*	1 (0.3%)
*Mucor* spp.	1 (0.3%)
*Rhizopus orizae*	1
*Kodamaea ohmeri*	1 (0.3%)

**Table 3:** Differences between histoplasmosis and other invasive fungal infections.

	Histoplasmosis	Other Invasive Fungal Infections	*p*
	N = 133	N = 119	
Men (N, %)	85 (64%)	75 (63%)	0.9
CD4 (median, [IQR])	62 (25–123)	57 (23–172)	0.9
Age at diagnosis (mean ± sd)	46.5 ± 12	46.3 ± 12	0.7
Country of birth			
Brazil (N,%)	32 (24%)	20 (16.8%)	0.01
Suriname (N,%)	25 (19%)	12 (10.1%)	0.01
France (N, %)	22 (17%)	26 (21.8%)	0.54
Haiti (N, %)	21 (16%)	35 (29.4%)	reference
Guyana (N, %)	6 (5%)	7 (5.9%)	0.78
Other (N, %)	28 (21%)	19 (16%)	0.2

## Data Availability

Data on HIV patients being sensitive, upon reasonable request, and Commission Nationale Informatique et Libertés’ approval that the data may be shared.
